# Resting-State Activity of Prefrontal-Striatal Circuits in Internet Gaming Disorder: Changes With Cognitive Behavior Therapy and Predictors of Treatment Response

**DOI:** 10.3389/fpsyt.2018.00341

**Published:** 2018-08-03

**Authors:** Xu Han, Yao Wang, Wenqing Jiang, Xiaochen Bao, Yawen Sun, Weina Ding, Mengqiu Cao, Xiaowei Wu, Yasong Du, Yan Zhou

**Affiliations:** ^1^Department of Radiology, Ren Ji Hospital, School of Medicine, Shanghai Jiao Tong University, Shanghai, China; ^2^Department of Child and Adolescent Psychiatry, Shanghai Mental Health Center, Shanghai Jiao Tong University, Shanghai, China

**Keywords:** internet gaming disorder, cognitive behavior therapy, functional magnetic resonance imaging, amplitude of low-frequency fluctuation, functional connectivity

## Abstract

Cognitive behavior therapy (CBT) is effective for the treatment of Internet gaming disorder (IGD). However, the mechanisms by which CBT improves IGD-related clinical symptoms remain unknown. This study aimed to discover the therapeutic mechanism of CBT in IGD subjects using resting-state functional magnetic resonance imaging (rsfMRI). Twenty-six IGD subjects and 30 matched healthy controls (HCs) received rsfMRI scan and clinical assessments; 20 IGD subjects completed CBT and then were scanned again. The amplitude of low-frequency (ALFF) values and the functional connectivity (FC) between the IGD group and the HC group were compared at baseline, as well as the ALFF values and FC before and after the CBT in the IGD group. Prior to treatment, the IGD group exhibited significantly increased ALFF values in the bilateral putamen, the right medial orbitofrontal cortex (OFC), the bilateral supplementary motor area (SMA), the left postcentral gyrus, and the left anterior cingulate (ACC) compared with the HC group. The HC group showed significantly increased FC values between the left medial OFC and the putamen compared with the IGD group, the FC values of IGD group were negatively associated with the BIS-11 scores before treatment. After the CBT, the weekly gaming time was significantly shorter, and the CIAS and BIS-II scores were significantly lower. The ALFF values in the IGD subjects significantly decreased in the left superior OFC and the left putamen, and the FC between them significantly increased after the CBT. The degree of the FC changes (Δ_FC/Pre−FC_) was positively correlated with the scale of the CIAS scores changes (Δ_CIAS/Pre−CIAS_) in the IGD subjects. CBT could regulate the abnormal low-frequency fluctuations in prefrontal-striatal regions in IGD subjects and could improve IGD-related symptoms. Resting-state alternations in prefrontal-striatal regions may reveal the therapeutic mechanism of CBT in IGD subjects.

## Introduction

Internet gaming disorder (IGD), also known as problematic Internet use, is the excessive and recurrent use of online Internet games (1). More recently, IGD was listed as the persistent or recurrent gaming behavior characterized by an impaired control over gaming; increased priority given to gaming over other activities to the extent that gaming takes precedence over other interests and daily activities; and the continuation of gaming despite the occurrence of negative consequences (2, 3). Although no formal diagnostic criteria for a psychiatric condition characterized by excessive and interfering patterns of Internet use were included in the fourth edition of the Diagnostic and Statistical Manual (DSM-IV) (4), the DSM-V committee is considering using the generated criteria for substance -use and addictive disorders for IGD and has included IGD in the section denoting further investigation (5).

Researchers have likened IGD to impulse-control disorders (6). Neuroimaging studies found that excessive Internet gaming was associated with abnormal resting-state activity in the frontal lobe, the brain region responsible for cognitive process, such as inhibitory control (7). Impaired function of the prefrontal (PFC) may relate to high impulsivity, which, in turn may contribute to the impaired inhibitory control associated with IGD (8). Effective cognitive control is associated with the coordinated recruitment of different top-down, prefrontal-striatal circuits (9, 10). Previous studies revealed the association between structural and functional abnormalities in the prefrontal cortex (PFC) and impaired inhibitory control in IGD (11–16). For example, reduced cortical thickness and an increased amplitude of low frequency fluctuation (ALFF) value in the OFC were found to be correlated with the impairment of the cognitive control function in young subjects with IGD (12). A study using the Reho method found that IGD subjects showed increased synchronization in the superior frontal gyrus compared to healthy controls (HCs), which suggested an increase in the neural activity associated with cognitive control function (17). Ko et al. (10) demonstrated that the impaired function in prefrontal-striatal regions may explain the decrease in the inhibitory capacity in IGD. These imaging studies characterized how both frontal lobe structures and functions are altered in association with impaired inhibitory control in IGD. Furthermore, impaired dopamine function in the striatum (a decrease in dopamine D2 receptors and reduced dopamine release) and its association with reduced baseline glucose metabolism in the PFC were observed (18, 19).

Cognitive behavior therapy (CBT) has been found to be effective in treating impulse control disorders, including pathological gambling (20). Studies of substance addiction have indicated that CBT encourages subjects to recognize and avoid situations in which they may be likely to use substances and to use coping strategies to resist drug use and improve inhibitory control function (21, 22). A study using the Stroop task found that CBT may be associated with a reduction in substance use, and it may affect the neural systems involved in cognitive control, impulsivity, motivation, and attention (23). Another functional magnetic resonance imaging (fMRI) study that employed a monetary incentive delay (MID) task in cannabis dependence reported that cannabis-dependent participants demonstrated decreased bilateral putamen volumes following CBT, which indicated that the specific aspects of putamen function and structure relate to treatment outcomes (24). Young believes that the intervention in Internet Addiction (IA) should focus on the restraint of Internet use, based on this, he proposes cognitive behavior therapy-IA (CBT-IA) approach, which has been proved to be effective on the treatment of IGD (6). Dr Du' s group found that school-based group CBT is effective for adolescents with IGD, particularly in improving emotional state and regulation ability, behavioral and self-management style (20). Though CBT has demonstrated considerable efficacy in the treatment of IGD, few studies have investigated the therapeutic mechanism of CBT in IGD subjects using fMRI. Investigation of the brain changes before and after treatment can not only improve our understanding of the pathogenesis of IGD and the therapeutic mechanism of CBT on IGD, but can also help to monitor treatment effects.

We used the Barratt Impulsiveness Scale-11 (BIS-11) to assess the behavioral inhibition function of IGD. Based on previous studies, we hypothesized that (1) subjects with IGD may show abnormal brain activity /connectivity in prefrontal-striatal regions, which are responsible for cognitive process, such as inhibitory control; (2) CBT could regulate the abnormal function of prefrontal-striatal regions.

## Materials and methods

### Participants and clinical assessments

The current study was approved by the Research Ethics Committee of Ren Ji Hospital and School of Medicine, Shanghai Jiao Tong University, China No. [2016] 097k (2). All participants and guardians signed written informed consent forms prior to the study. The enrolled participants, the diagnostic questionnaire and the exclusion criteria were all described in our previous publication (15). Twenty-six IGD subjects who met the standards of the Diagnostic Questionnaire for Internet Addiction (i.e., YDQ) test modified by Beard and Wolf (25) were recruited from the Department of Child and Adolescent Psychiatry of the Shanghai Mental Health Center. Thirty age- and gender-matched healthy individuals with no personal or family history of psychiatric disorders were recruited as the healthy control (HC) group through advertisements. Given the higher prevalence of IGD in men vs. women, only male participants were included (26). All participants were right-handed, and none of them smoked.

All participants underwent a simple physical examination, which included blood pressure and heart rate measurements, and were interviewed by a psychiatrist regarding their medical history of nervous, motor, digestive, respiratory, circulatory, endocrine, urinary, and reproductive problems. They were then screened for psychiatric disorders with the Mini International Neuropsychiatric Interview for Children and Adolescents (MINI-KID) (27). The exclusion criteria were a history of substance abuse or dependence; previous hospitalization for psychiatric disorders; or a major psychiatric disorder, such as schizophrenia, depression, anxiety disorder, and/or psychotic episodes.

A basic information questionnaire was used to collect demographic information such as gender, age, final year of schooling completed, and hours of Internet use per week. Four questionnaires were used to assess the participants' clinical features, namely, the Chen Internet Addiction Scale (CIAS) (28), the Self-rating Anxiety Scale (SAS) (29), the Self-rating Depression Scale (SDS) (30), and the Barratt Impulsiveness Scale-11 (BIS-11) (31). The CIAS, developed by Chen, contains 26 items on a four-point Likert scale and reflects the severity of Internet addiction. The SAS and SDS were used to show that all the subjects meet the inclusion criteria during the research period. All questionnaires were initially written in English and then were translated into Chinese. Then, 26 IGD subjects, their parents and their teachers participated in the follow-up group CBT voluntarily, which consists 12 sessions (20). Each session lasted 1.5–2 h. In each session of group therapy, a different topic was discussed. These topics included how to recognize and control your feelings; principles of healthy communication between parents and children; techniques for dealing with relationships developed via the Internet; techniques for dealing with content experienced via the Internet; techniques for controlling your impulses; techniques for recognizing when addictive behavior is occurring; and how to stop addictive behavior. The last session was a review session.

Following the intervention, we assessed the clinical characteristics of the IGD subjects again, and twenty of them were scanned once more on a voluntary basis in a similar way to that of the pre-CBT protocol.

### MR data acquisition

All subjects underwent resting-state fMRI at baseline with a 3.0-T MR imaging system (GE Signa HDxt3T, USA) with a standard head coil. To avoid motion and to reduce scanner noise, soft pads were used, and the subjects were given thorough instructions to void moving during the scan and explanations as to why motion is not preferable, in addition to the instructions that excessive motion would lead to a rescan. Resting-state fMRI data were acquired using a gradient-echo echo-planar sequence as described in our previous study (16). Thirty-four transverse slices [repetition time [TR] = 2,000 ms; echo time [TE] = 30 ms; field of view [FOV] = 230 × 230 mm; and 3.6 × 3.6 × 4 mm voxel size] covering the entire brain were obtained along the anterior commissure-posterior commissure line. For this scan sequence, 220 functional volumes were obtained while the subjects were resting (resulting in a scan length of 440 s). During the scanning, the participants were instructed to keep still with their eyes closed, as motionless as possible, and not to sleep or think about anything. After the scanning, the subjects were asked to confirm whether they remained awake during the scan. Another two sequences were also acquired: (1) an axial T1-weighted fast spin-echo sequence (TR = 1,725 ms; TE = 24 ms; FOV = 256 × 256 mm; 34 slices; and 0.5 × 0.5 × 4 mm voxel size) and (2) an axial T2-weighted fast spin-echo sequence (TR = 9,000 ms; TE = 120 ms; FOV = 256 × 256 mm; 34 slices; and 0.5 × 0.5 × 4 mm voxel size).

### Preprocessing of functional imaging data

Preprocessing of the imaging data was performed using SPM12 implemented in MATLAB and SPM12's extension software Data Processing and Analysis of Brain Imaging (DPABI; http://rfmri.org/dpabi) (32). After discarding the first 10 volumes of each functional time series, the remaining 210 images were slice-time corrected, realigned to the middle volume, and realigned by using a six-parameter (rigid body) linear transformation. Then, all the functional images were directly normalized to the EPI template, each voxel was resampled to 3 × 3 × 3 mm, and a spatial smoothing transformation was conducted with an 8-mm full-width half-maximum Gaussian kernel. Then, the 26 nuisance covariates (including the mean time course of the signals from the voxels within the white matter mask, the mean time course of signals from the voxels within the CSF mask, and the Friston 24 motion parameters) were regressed out. In addition, the linear trend was included as a regressor since the BOLD signal can demonstrate low-frequency drift.

No participant in this study exhibited movement greater than 1.5 mm of maximum translation in the *x, y*, or *z* axes or a maximum rotation of 1.5° in any of the 3 axes. To further rule out the residual effect of motion on the resting-state fMRI measures, the mean framewise displacement (mean FD) of the head motion was computed and used as a covariate in all voxelwise group functional analyses, which were derived with Jenkinson's relative root mean square algorithm and considered the voxelwise differences in motion in its derivation (33); no group differences were found in the mean FD between the IGD and HC subjects (*p* = 0.52) at baseline or between the pre-CBT and post-CBT timepoints (*p* = 0.71).

### Functional imaging data analysis

The ALFF analyses were performed using the DPABI software. The ALFF is proportional to the strength or intensity of low-frequency oscillations and is thought to reflect spontaneous neural activity (34, 35). In brief, after the previously mentioned preprocessing, the time series of each voxel was transformed to the frequency domain without bandpass filtering, and the power spectrum was obtained. Then, the power spectrum was square root transformed and averaged across 0.01–0.08 Hz at each voxel. The averaged square root of power in this frequency band was taken as the ALFF value. Then, with a standardization procedure, each individual ALFF map was normalized by the individual's global mean ALFF; more specifically, the mean across the voxels of the ALFF map was calculated, and the value of each voxel was divided by the mean individually. We first compared the baseline ALFF of the IGD group with that of the HC group to explore the altered neural activity in the IGD subjects by means of a two-sample *t-*test. A correction for multiple comparisons resulting in a corrected threshold of *p* < 0.05 was implemented, with a minimum cluster size of 42 voxels (AlphaSim-corrected with the following parameters: single voxel *p* = 0.001; 5,000 simulations; a mean estimated spatial correlation of 8.04 × 10.60 × 10.46 mm FWHM; and the global gray matter mask). To examine the effects of the CBT on the IGD subjects, a paired *t*-test was performed to compute the ALFF group difference map before and after CBT. A correction for multiple comparisons resulting in a corrected threshold of *p* < 0.05 was implemented, with a minimum cluster size of 40 voxels (AlphaSim-corrected with the following parameters: single voxel *p* = 0.001; 5,000 simulations; a mean estimated spatial correlation of 9.70 × 10.30 × 9.52 mm FWHM; and the global gray matter mask). The smoothing kernel was estimated based on the t map. The coordinates of the regions with significant group differences are reported in the Montreal Neurologic Institute (MNI) space.

The regions of interest (ROIs) were determined to be the regions where the ALFF values changed significantly between the pre- and post-CBT time points. The FC values of the seed regions (the left superior OFC (MNI coordinates: x = −12, y = 24, z = −21, radius = 6 mm) and the left putamen (MNI coordinates: x = −3, y = 3, z = 9, radius = 6 mm) were extracted using DPABI. At baseline, a two-sample *t*-test was used to compare the FC values between the IGD group and HC group and Pearson correlation analyses were conducted between the FC values and the scores of CIAS / BIS-11 in IGD group. Then a paired *t-*test was used to compare the FC values between the pre- and post-treatment time points. Pearson correlation analyses were conducted between the degree of change in the extracted FC values (Δ_ALFF/Pre−ALFF_ or Δ_FC/Pre−FC_) and the scale of the reduction in the CIAS scores (Δ_CIAS/Pre−CIAS_) /BIS-11 (Δ_BIS−11/Pre−BIS−11_) scores to investigate whether FC changes would predict symptom reduction through CBT, according to the methods described in the previous study (36). A two-tailed *p*-value of 0.05 was considered statistically significant.

### Statistical analysis of demographic and clinical measures

Two-sample *t-*tests were carried out using SPSS (Statistical Package for the Social Sciences software, SPSS version 19, IBM, USA) for the continuous variables to assess the differences between the IGD group and the HC group. Paired *t*-tests were used to examine the effects of CBT on the clinical characteristics between the pre- and post-CBT timepoints.

## Results

### Demographics and clinical measures of the IGD and HC subjects

The IGD and HC subjects did not differ in either age (*p* = 0.31) or education (*p* = 0.10). As expected, the IGD subjects showed significantly higher CIAS, SAS, SDS, and BIS-II scores (*p* < 0.001, *p* = 0.02, 0.04, 0.001), as well as longer weekly gaming time than the HC subjects did (*p* < 0.001; Table 1).

**Table 1:** Demographic and behavioral characteristics of the IGD and HC group.

	**IGD (*n* = 26)**	**HC (*n* = 30)**	***P*-value**
	**(Mean ± SD)**	**(Mean ± SD)**	
Age(yeas)	16.81 ± 0.75	17.00 ± 0.89	0.31
Education (yeas)	11.53 ± 0.70	11.20 ± 0.81	0.10
Time for internet use per week (hours)	32.54 ± 10.34	1.70 ± 5.36	<0.001
Chen Internet Addiction Scale (CIAS)	71.88 ± 5.56	41.97 ± 11.31	<0.001
Self-Rating Anxiety Scale (SAS)	45.65 ± 10.24	40.10 ± 7.28	0.02
Self-rating depression scale (SDS)	48.23 ± 8.34	43.43 ± 8.97	0.04
Barratt Impulsiveness Scale-11 (BIS-11)	59.62 ± 9.11	52.27 ± 6.90	0.001

*SD, Standard deviation; IGD, internet gaming disorder; HC, healthy control; CBT, cognitive behavior therapy*.

### ALFF and FC differences between the IGD and HC subjects

**Figure 1:**
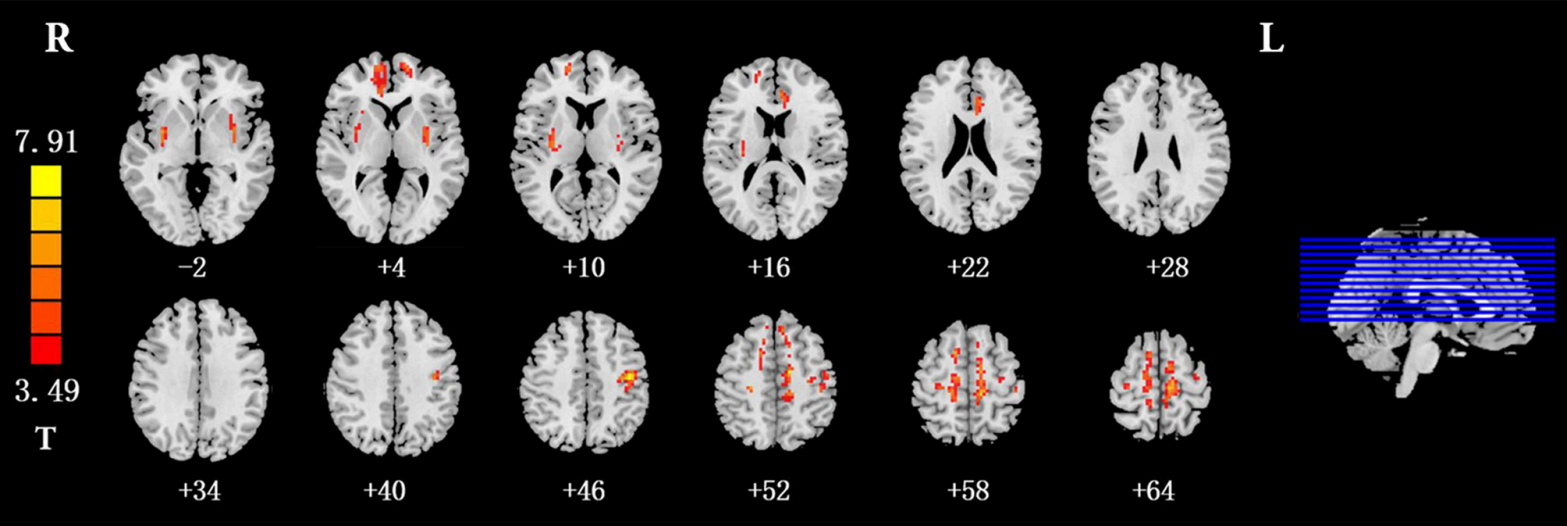
Brain regions that showed higher ALFF values in the IGD group than in the HC group at baseline (*p* < 0.05, AlphaSim-corrected). The left part of the figure represents the participant's right side, and the right part represents the participant's left side. ALFF, amplitude of low frequency fluctuation; IGD, internet gaming disorder; HC, healthy control.

Compared with the HC subjects, the IGD subjects showed significantly increased ALFF values in the bilateral putamen, the right medial OFC, the bilateral supplementary motor area (SMA), the left postcentral gyrus, and the left anterior cingulate (ACC; Table 2, Figure 1). The resting-state FC between the left medial OFC and the putamen was significantly lower in IGD group (*p* = 0.002).

**Table 2:** Regions showing group differences on ALFF between IGD group and HC group.

**Cluster description**	**BA**	**MNI coordinates**			**Cluster size**	**Peak *t* score**
		**X**	**Y**	**Z**		
Putamen (L)		−33	0	−3	95	6.02
Putamen (R)		33	3	−3	56	5.19
Medial orbitofrontal cortex (R)	11	12	60	3	214	5.33
Supplementary motor area (L)	6	−12	−7	56	464	7.21
Postcentral gyrus (L)	6	−42	−15	45	103	7.91
Anterior cingulate (L)	24	−6	14	31	62	6.26
Supplementary motor area (R)	6	12	9	57	276	6.16

*BA, Brodmann area; IGD, internet gaming disorder; HC, healthy control. Two sample-T test P < 0.05, AlphaSim-corrected (P < 0.001, voxel size>42)*.

### Demographics and clinical measures before and after CBT

After the CBT, the weekly gaming time and the score of the CIAS and the BIS-11 were significantly reduced (all *p*s = 0.001). These findings indicated that the CBT was effective on the treatment of the IGD subjects (Table 3).

**Table 3:** Demographic and behavioral characteristics before and after cognitive behavior therapy (CBT) in IGD group.

	**Pre-CBT (*n* = 26)**	**Post-CBT (*n* = 26)**	***P*-value**
	**(Mean ± SD)**	**(Mean ± SD)**	
Time for internet use per week (hours)	32.54 ± 10.34	27.27 ± 9.36	0.001
Chen Internet Addiction Scale (CIAS)	71.88 ± 5.56	50.00 ± 11.99	0.001
Self-Rating Anxiety Scale (SAS)	45.65 ± 10.24	44.65 ± 10.24	0.630
Self-rating depression scale (SDS)	48.23 ± 8.34	46.77 ± 9.89	0.500
Barratt Impulsiveness Scale-11 (BIS-11)	59.62 ± 9.11	52.69 ± 10.04	0.001

*SD, Standard deviation; IGD, internet gaming disorder*.

### Changes in resting-state neural activity before and after CBT

After the CBT, the ALFF values was significantly decreased in the left medial OFC and the putamen (Table 4, Figure 3). In addition, the resting-state FC between the left medial OFC and the putamen was significantly increased.

**Table 4:** Regions showing group differences on ALFF between pre-CBT and post-CBT in IGD group.

**Cluster description**	**BA**	**MNI coordinates**			**Cluster size**	**Peak *t* score**
		**X**	**Y**	**Z**		
The superior orbitofrontal cortex (L)	11	−12	24	−21	41	−5.18
Putamen (L)		−15	12	−4	68	−6.19

BA, Brodmann area; CBT, cognitive behavior therapy, IGD, internet gaming disorder

*Paired-T test P < 0.05, AlphaSim-corrected (P < 0.001, voxel size>40)*.

### Clinical measures relationships

**Figure 2:**
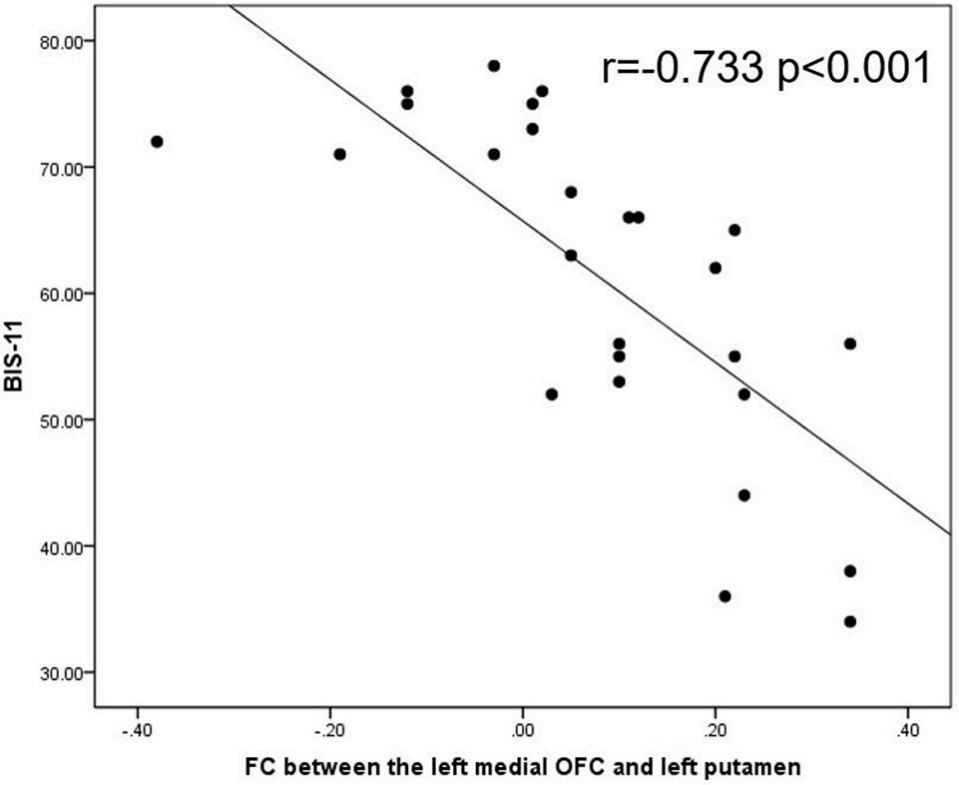
In the IGD group, the FC values between the left medial OFC and the putamen were negatively associated with the BIS-11 scores (*r* = −0.733, *p* < 0.001). IGD, Internet gaming disorder; FC, functional connectivity; OFC, orbitofrontal cortex; BIS-11, Barratt Impulsiveness Scale-11.

In the IGD group, the FC values between the left medial OFC and the putamen were negatively associated with the BIS-11 scores (*r* = −0.733, *p* < 0.001; Figure 2). The changes in the extracted FC values (Δ_FC/Pre−FC_) between the left superior OFC and the left putamen were positively correlated with the scale of the reduction in the CIAS scores (Δ_CIAS/Pre−CIAS_; *r* = 0.707, *p* < 0.001; Figure 4). No significant correlation between the changes of FC values (Δ_FC/Pre−FC_) and the scale of the reduction in the BIS-11 scores (Δ_BIS−11/Pre−BIS−11_) was detected (*r* = 0.396, *p* = 0.084).

**Figure 3:**
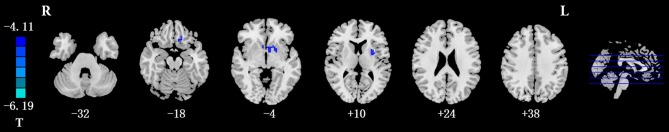
Brain regions that showed decreased ALFF values in the IGD group after the cognitive behavior therapy (*p* < 0.05, AlphaSim-corrected). The left part of figure represents the participant's right side, and the right part represents the participant's left side. IGD, Internet gaming disorder; ALFF, amplitude of low frequency fluctuation.

**Figure 4:**
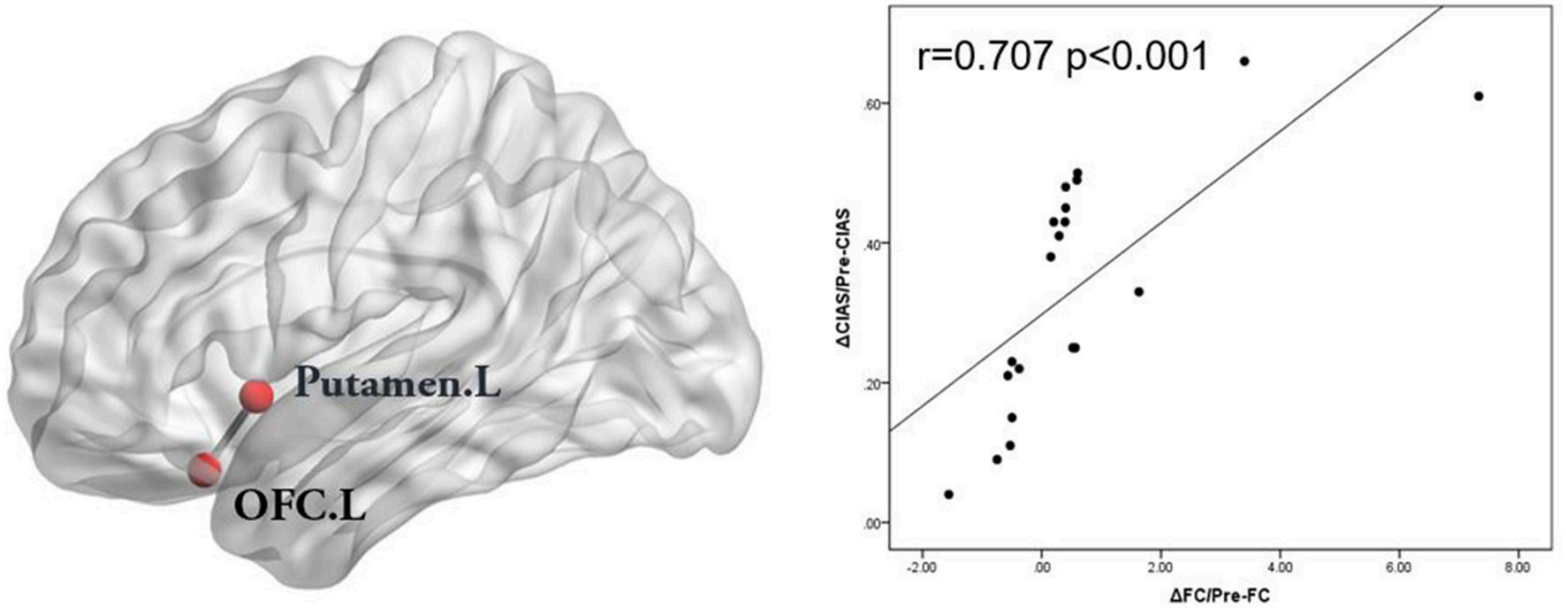
The changes in the FC values (ΔFC/Pre-FC) between the left superior OFC and the left putamen were positively correlated with the scale of reduction in the CIAS scores in the IGD subjects. (ΔCIAS/Pre-CIAS; *r* = 0.707, *p* < 0.001). FC, functional connectivity; OFC, orbitofrontal cortex; CIAS, Chen Internet Addiction Scale; IGD, Internet gaming disorder.

## Discussion

In this longitudinal study, the ALFF and FC method were employed to investigate functional brain alternations between IGD group and HC group and the therapeutic mechanism of CBT in IGD subjects. We found that IGD subjects demonstrated abnormal function of some prefrontal-striatal regions relative to the HC subjects and that CBT could attenuate the functional abnormalities in the OFC and the putamen and increase the interactions between them, in addition to improving the symptoms of IGD.

In this study, the resting-state FC between the left medial OFC and the putamen was significantly lower in IGD group. The BIS-11 correlates of the FC alternations demonstrated that the impairment in the prefrontal-striatal circuits may have an impact on impulsive behavior of IGD subjects. Previous neuroimaging studies reported that functional impairment in the PFC regions was associated with the high impulsivity in IGD (37). The prefrontal-striatal circuits include a cognitive loop, which mainly connects the caudate and the putamen with prefrontal regions. Consistent with the findings of recent functional neuroimaging studies, functional alternations were observed in several prefrontal regions (including the right medial OFC, the bilateral SMA and the left ACC) and basal ganglia regions (the bilateral putamen) in addictive disorders, including IGD (12, 38, 39). Volkow et al. suggested neuronal networks in drug-addicted subjects, including the OFC-, ACC-, inferior frontal gyrus (IFG)-, and dorsolateral prefrontal cortex (DLPFC)-striatal circuits, that may reflect observable behaviors, such as impaired self-control and behavior inflexibility (40) and problems in making good decisions, that characterize the addiction; when individuals with IGD continue to play games even though they are confronted with negative consequences, this might be related to the impaired function of prefrontal-striatal circuits (41). One of the core behaviors of IGD is impulse control deficits with a lack of control over Internet gaming playing. A previous study combining voxel-based morphometric (VBM) and FC analyses revealed the involvement of several prefrontal regions and the related prefrontal-striatal circuits (ACC-, OFC-, and DLPFC-striatal circuits) in the process of IGD and suggested that IGD may share similar neural mechanisms with substance dependence at the circuit level (41). The current finding is important, as the alternations of brain activity/connectivity in prefrontal-striatal circuits that were observed dovetails with previous studies. In addition, the SMA is included in salience network, which regulates the function of other networks when rapid changes in behavior are required, such as when quickly manipulating the keyboard while playing games (42). Yuan et al. reported higher ALFF values in the SMA in IGD subjects (12), and we found a similar result in this study, which suggested that the SMA may be a potentially important region in addictive behaviors (41).

To date, group CBT has been shown to be effective in aiding adolescents with Internet addiction (20). In the present study, weekly gaming time was significantly shorter, and the scores of the CIAS and the BIS-II were significantly reduced after the CBT. It suggested that the negative consequences could be reversed if Internet addiction could be remitted within a short duration. We observed decreased ALFF values in the left superior OFC and the left putamen and the increased OFC-putamen connectivity after the CBT, which are findings that are consistent with previous observations that suggested that the OFC-striatal circuit may be a potential therapeutic target across addictive disorders (43). The OFC is involved in impulse regulation in addition to decision making, so the connectivity between the OFC and the putamen imply a better control over impulsive behavior of IGD subjects (44). It is consistent with the result of reduced BIS-11 scores after treatment. The putamen is one of the sectors of the striatum and has been a brain region associated with cognitive processes that are largely shared with the caudate nucleus. More specifically, the putamen has been associated with control of habitual behaviors and goal-directed actions (45). We observed that the higher ALFF decreased in the left putamen after CBT, suggesting that CBT may be helpful in enhancing the control of the habitual behaviors and goal-directed actions of IGD subjects. This means that CBT may be able to prevent habitual emotionless game use by changing the interactions of the prefrontal-striatal circuits. Previous studies of CBT have reported that CBT alters resting-state activation in the prefrontal cortex and that CBT corrects dysfunctional cognitive processes (46). Meanwhile, the changes in the OFC-putamen connectivity could predict the effect of CBT.

A weakness of this study was that the IGD subjects were not randomly assigned to two groups (one group of the participants would receive the CBT, while another group who did not receive the treatment would serve as a control). Second, we recruited only male participants; thus, further studies with female participants are needed to confirm and extend the current results. Third, the limited sample size increased the risk of false negatives and constrained the test to evaluating relationships between the changes in the FC values and the treatment effects. Fourth, it is necessary to correct for multiple comparisons to control the false-positive error. AlphaSim correction was used here because no cluster can be obtained when using the FWE or FDR correction methods. However, we think the AlphaSim correction can be accepted in our exploratory study since it is one of the most popular choices for multiple-comparisons correction and used in many studies (34).

In summary, our findings showed that IGD was associated with altered function of some prefrontal-striatal circuits and that CBT could both attenuate the functional abnormalities of the OFC and the putamen and increase the interactions between them. These findings may provide a basis for revealing the therapeutic mechanism of CBT in IGD subjects and serve as potential biomarkers that may predict symptom improvement following CBT in IGD subjects.

## Author contributions

YZ, YD were responsible for the study concept and design. YD, WJ, XB, MC, XW, and WD contributed to the acquisition of data. YS, XH, and YW assisted with data analysis and interpretation of findings. XH drafted the manuscript. All authors critically reviewed content and approved final version for publication.

### Conflict of interest statement

The authors declare that the research was conducted in the absence of any commercial or financial relationships that could be construed as a potential conflict of interest.
